# Systemically administered PEDF against primary and secondary tumours in a clinically relevant osteosarcoma model

**DOI:** 10.1038/bjc.2011.410

**Published:** 2011-10-06

**Authors:** M L Broadhead, C R Dass, P F M Choong

**Affiliations:** 1 Department of Orthopaedics, St Vincent's Hospital Melbourne, Level 3, Daly Wing, 35 Victoria Pde, Fitzroy, VIC 3065, Australia; 2 University of Melbourne, Department of Surgery, St Vincent's Hospital Melbourne, Level 2, Clinical Sciences Building, 29 Regent St Fitzroy, VIC 3065, Australia; 3 School of Biomedical and Health Sciences, Victoria University, St Albans, VIC 3021, Australia; 4 Sarcoma Service, Peter MacCallum Cancer Centre, East Melbourne, VIC 3002, Australia

**Keywords:** PEDF, osteosarcoma, orthotopic model

## Abstract

**Background:**

Pigment epithelium-derived factor (PEDF) is an endogenous glycoprotein with a potential role as a therapeutic for osteosarcoma. Animal studies have demonstrated the biological effects of PEDF on osteosarcoma; however, these results are difficult to extrapolate for human use due to the chosen study design and drug delivery methods.

**Methods:**

In this study we have attempted to replicate the human presentation and treatment of osteosarcoma using a murine orthotopic model of osteosarcoma. The effects of PEDF on osteosarcoma cell lines were evaluated *in vitro* prior to animal experimentation. Orthotopic tumours were induced by intra-tibial injection of SaOS-2 osteosarcoma cells. Treatment with PEDF was delayed until after the macroscopic appearance of primary tumours. Pigment epithelium-derived factor was administered systemically via an implanted intraperitoneal micro-osmotic pump.

**Results:**

*In vitro*, PEDF inhibited proliferation, induced apoptosis and inhibited cell cycling of osteosarcoma cells. Pigment epithelium-derived factor promoted adhesion to Collagen I and inhibited invasion through Collagen I. *In vivo*, treatment with PEDF caused a reduction in both primary tumour volume and burden of pulmonary metastases. Systemic administration of PEDF did not cause toxic effects on normal tissues.

**Conclusion:**

Systemically delivered PEDF is effective in suppressing the size of primary and secondary tumours in an orthotopic murine model of osteosarcoma.

Osteosarcoma is an aggressive primary bone cancer that predominately affects adolescents and young adults. Neo-adjuvant chemotherapy, adjuvant chemotherapy and surgical resection are the mainstays of treatment for osteosarcoma. Advances in diagnostic imaging have provided a more complete evaluation of tumour anatomy and have allowed the treating surgeon to consider a variety of limb salvage techniques. Despite these advances, however, patient prognosis has not improved significantly since the 1970s when multiagent chemotherapy regimes were introduced (Guise *et al*, 2009). The 5-year survival rate for osteosarcoma remains steady at 60–70% (Kumar *et al*, 2005). Novel approaches are desperately needed to improve the treatment of patients with osteosarcoma, particularly for those with chemoresistant or recurrent disease.

With this challenge in mind, research has focused on characterising the genetic basis of osteosarcoma. The molecular pathways that underlie tumourigenesis, proliferation, invasion and metastasis are being identified as targets for novel treatment agents (Broadhead *et al*, 2010). Targeting the deranged molecular signalling of osteosarcoma should enhance the effectiveness of conventional chemotherapeutics and possibly reduce patient morbidity.

Pigment epithelium-derived factor (PEDF) is a multifunctional molecule with a potential role as a therapeutic agent for osteosarcoma. Pigment epithelium-derived factor is an endogenous 50-kDa glycoprotein that was first shown to be capable of inducing differentiation of Y-79 retinoblastoma cells (Tombran-Tink and Johnson, 1989). Pigment epithelium-derived factor is expressed in a wide range of tissues including the eye, brain, spinal cord, plasma, bone, cartilage, heart, lung, prostate and pancreas (Broadhead *et al*, 2009). Pigment epithelium-derived factor has diverse roles in these tissues; however, it has attracted attention foremost as a potent anti-angiogenic agent. Pigment epithelium-derived factor is twice as potent as angiostatin and seven times as potent as endostatin (Dawson *et al*, 1999). It was the anti-angiogenic properties of PEDF that lead to the study of its potential as an anti-tumour agent for various cancers (Broadhead *et al*, 2009). Anti-angiogenic agents such as bevacizumab have already been adopted as adjunctive treatments for cancers, including metastatic colon carcinoma, breast carcinoma, renal cell carcinoma, non-small-cell lung carcinoma and glioblastoma multiforme.

Previous studies have provided proof of principle for PEDF as an anti-osteosarcoma agent. Quan *et al* (2002) first examined the role of cartilage-derived anti-angiogenic factors at the growth plate of long bones. Using immunohistochemistry and *in situ* hybridisation, PEDF expression was shown to be largely restricted to the avascular resting, proliferative and upper hypertrophic layers of the growth plate. These are the regions that are consistently resistant to osteosarcoma invasion from the adjacent metaphysis. Ek *et al* (2007a) and Takenaka *et al* (2005) later showed that PEDF restricted osteosarcoma growth *in vitro* through both the induction of apoptosis and the inhibition of cell cycling. Pigment epithelium-derived factor also restricted the metastatic capacity of osteosarcoma cells by improving cellular adhesion and restricting invasion.

Pigment epithelium-derived factor has been tested in a number of compelling *in vivo* animal studies for osteosarcoma. Ek *et al* (2007a) applied PEDF to a spontaneously metastasising orthotopic model of osteosarcoma. SaOS-2 human osteosarcoma cells were first treated with PEDF and primary osteosarcoma was induced by intra-tibial injection of treated cells in Balb/c nude mice. Pigment epithelium-derived factor restricted the growth of primary tumours and the occurrence of pulmonary metastases. Ek *et al* (2007b) also showed that PEDF overexpression in an orthotopic model reduced microvessel density and osteolysis. Pigment epithelium-derived factor gene delivery in this model resulted in reduced tumour growth, both when used alone and in combination with doxorubicin therapy (Ta *et al*, 2009a).

All of these previous *in vivo* studies with PEDF have utilised a clinically relevant orthotopic model that allows an evaluation of both primary and secondary tumour progression. However, while showing proof of principle, the results of treating osteosarcoma cells with PEDF prior to inoculation (Ek *et al*, 2007a), and the use of a PEDF-expressing plasmid (Ek *et al*, 2007b; Ta *et al*, 2009a), are difficult to extrapolate for human use. In order to truly evaluate the therapeutic efficacy of PEDF in a clinically relevant model of disease, treatment with PEDF should be delayed until after the establishment of primary tumours, and preferably be performed with systemic recombinant protein. This would more accurately replicate the human condition where patients most commonly present for treatment with an established tumour.

In this study we aimed to evaluate systemically administered PEDF in a model optimised for clinical relevance. Using an orthotopic murine model of spontaneously metastasising osteosarcoma, we show for the first time that systemic delivery of PEDF is capable of restricting the size of established primary and secondary osteosarcoma.

## Materials and methods

### Cells, culture conditions, reagents and mice

SaOS-2 human osteosarcoma cells were obtained from the American Tissue Culture Collection (ATCC, Manassas, VA, USA). SJSA-1 human osteosarcoma cells were kindly provided by A/Professor David Thomas (Peter MacCallum Cancer Centre, East Melbourne, Australia). Cells were cultured in complete medium (CM) under standard conditions at 37°C and in humidified 5% CO_2_. Complete medium consisted of MEM-Alpha+GlutaMAX (Invitrogen, Carlsbad, CA, USA) supplemented with 10% fetal bovine serum (Invitrogen) and 1% antibiotic-antimycotic (Invitrogen).

Five-week-old Balb/c nude mice were purchased from the Animal Resource Centre, Australia, and were housed at the St Vincent's Hospital BioResources Centre. The St Vincent's Hospital Melbourne Animal Ethics Committee approved all animal experimentation.

Pigment epithelium-derived factor was obtained from BioProducts MD (Middletown, MD, USA).

### MTS proliferation assay

Cell lines SaOS-2 and SJSA-1 were seeded in 96-well plates at a density of 1 × 10^3^ cells per well, 100 *μ*l per well in quadruplicate. Cells were cultured in CM for 24 h under standard conditions and then treated with PEDF (0, 1.56, 3.125, 6.25, 12.5, 25, 50, 100 nM). After 48 h treatment with PEDF, 20 *μ*l of CellTiter 96 AQ_ueous_ One Solution Reagent (Promega, Madison, WI, USA) was added to each well. This reagent contains the tetrazolium compound MTS (3-(4,5-dimethylthiazol-2-yl)-5-(3-carboxymethoxyphenyl)-2-(4-sulfophenyl)-2H-tetrazolium). Cells were incubated with the reagent for 1–3 h as per the manufacturer's instructions. Absorbance was measured hourly at 490/655 nm using a Bio-Rad Model 680 microplate reader (Bio-Rad, Philadelphia, PA, USA). This experiment was repeated four times, and representative experiments for SaOS-2 and SJSA-1 cell lines are presented here.

### Terminal deoxynucleotidyl transferase dUTP nick end labelling assay

Cell lines SaOS-2 and SJSA-1 were seeded in 96-well plates at a density of 1 × 10^3^ cells per well, 100 *μ*l per well in quadruplicate. After 24 h, cells were treated with 0 or 100 nM PEDF. After 48 h of treatment with PEDF, apoptotic cells were stained with a terminal dUTP nick end labelling (TUNEL) assay kit (Promega), according to the manufacturer's instructions. A representative field of each well in the plate was observed and photographed under × 10 objective. TUNEL-positive staining cells were counted. This experiment was repeated four times.

### Ki-67 immunocytochemistry

Cell lines SaOS-2 and SJSA-1 were seeded and treated in 96-well plates as for the preceding TUNEL assay in quadruplicate. Ki-67 immunocytochemistry was performed after 48 h of treatment with PEDF. Cells were first fixed with 4% paraformaldehyde at room temperature, then permeabilised with 0.3% saponin. Primary blocking with 2% rabbit serum/0.25% bovine serum albumin/0.1% saponin was performed for 30 min. Cells were then incubated overnight with a 1 : 50 dilution of monoclonal mouse anti-human Ki-67 antibody (DakoCytomation, Glostrup, Denmark). The following day, cells were incubated for 1 h at room temperature with 1 : 2000 diluted biotinylated polyclonal rabbit anti-mouse secondary antibody (DakoCytomation). A Vectastain ABC kit was then used according to the manufacturer's instructions and developed with SIGMA FAST DAB. Three drops of 100% glycerol was added to each well prior to microscopy, photography and enumeration under × 10 objective. This experiment was repeated four times.

### Collagen I adhesion assay

Collagen I (0.2%, BD Biosciences) was applied to the base of a 24-well plate and allowed to set at 37°C/5% CO_2_ for 60 min. Excess collagen was removed prior to seeding SaOS-2 and SJSA-1 cells at a density of 1 × 10^5^ per well in 500 *μ*l of CM±100 nM PEDF in duplicate. After 60 min at 37°C/5% CO_2_, each well was washed twice with PBS to remove loose cells and debris. Wells were observed under × 10 objective and photographed. Adherent cells were counted. This experiment was repeated three times.

### Collagen I invasion assay

Cell culture inserts with polyethylene terephthalate track-etched membranes, 8.0-*μ*m pore size, were inserted into 24-well plates, then coated with 2 mg ml^−1^ type I rat tail collagen (BD Biosciences). CM was placed in the wells beneath the cell culture insert. Cells SaOS-2 and SJSA-1 were seeded at 5 × 10^4^ cells per insert in serum-free medium in duplicate. Cells were incubated under standard conditions for 6 days. Polyethylene terephthalate membranes were then removed and prepared for microscopy using the QuickDip staining system. Membranes were imaged under × 20 objective and adherent cells were enumerated. The experiment was repeated twice.

### Electron microscopy

Cells (SaOS-2) were seeded and treated in 96-well plates according to the protocol used for TUNEL assay and Ki-67 immunocytochemistry. Cells were prepared for transmission electron microscopy (TEM) after 48 h of treatment with PEDF. Cells were fixed with 2.5% glutaraldehyde/0.1 M cacodylate buffer (pH 7.4) for 1 h and then post-fixed with 2.0% osmium tetroxide/deionised water for 1 h. Cells were dehydrated using a gradient of acetone, followed by infiltration with Spurr's resin and sectioning on an UltraCut-S microtome. Sections were stained with uranyl acetate/lead citrate solution. Transmission electron microscopy was performed on a Siemens 102 transmission microscope at 60 kV.

### Orthotopic model of osteosarcoma

Cells (SaOS-2) were mixed with 50% Matrigel to a concentration of 2 × 10^6^ cells ml^−1^. Mice were anaesthetised by intraperitoneal injection of 100 mg kg^−1^ ketamine and 10 mg kg^−1^ xylazine. A volume of 10 *μ*l of SaOS-2/Matrigel solution was injected into the left tibae of individual mice using a 27-gauge needle (Dass *et al*, 2006). The needle was inserted into the tibial tuberosity and advanced using a drilling motion to avoid fracture of the bone.

Mice were monitored thrice weekly for tumour growth and signs of distress. Tumours were measured in the anteroposterior (AP) and lateral (L) planes using digital callipers. Leg volume and tumour volume wearer calculated using the formula 4/3*π*(1/4(AP+L))^2^ (Ek *et al*, 2007a). The volume of the contralateral limb was subtracted from the tumour-bearing limb to calculate actual tumour volume. Mice were weighed using digital scales.

In this study, a total of 18 mice were injected with SaOS-2 cells initially. Variability of tumour take meant that of these 18, only 12 were suitable for randomisation to control and treatment groups. Tumours did not form in six mice. These groups, each consisting of four mice, received (1) sterile water as control, (2) PEDF 50 *μ*g kg^−1^ per day or (3) PEDF 500 *μ*g kg^−1^ per day. Sterile water was used as the PEDF diluent.

Sustained delivery of both sterile water and PEDF (BioProducts MD) was achieved by Alzet micro-osmotic pump (Durect Corp., Cupertino, CA, USA). The mean pumping rate for the Alzet micro-osmotic pump (model 1002) is 0.25 *μ*l h^−1^ over 14 days, as determined by the manufacturer. Pumps were implanted within the peritoneal cavities of mice at day 20 after SaOS-2 cell injection. The average tumour volume at this time was 21.1 mm^3^ (±2.357 s.e.m., *n*=12). Pumps remained *in situ* until the conclusion of the study at day 34.

Doses of PEDF (50 and 500 *μ*g kg^−1^) were selected based on published physiological and therapeutic concentrations. The physiological serum concentration of PEDF has previously been estimated at 100 nM (Petersen *et al*, 2003), while inhibition of vessel formation in ischaemia-induced retinopathy has been achieved at a 50 nM concentration (Stellmach *et al*, 2001). Reported serum PEDF concentrations for healthy human controls have since varied widely, ranging from 4 ng ml^−1^ to 15 *μ*g ml^−1^ (Matsumoto *et al*, 2004; Wiercinska-Drapalo *et al*, 2007; Nakamura *et al*, 2009; Sabater *et al*, 2010; Sogawa *et al*, 2011; Yang *et al*, 2011). The 50 and 500 *μ*g kg^−1^ doses used in this study are equivalent to 1 *μ*g ml^−1^ (20 nM) and 10 *μ*g ml^−1^ (200 nM) concentrations of PEDF, respectively, when the average mouse weight is taken as 20 g and the average blood volume 1 ml.

When tumours had grown to a disabling size for control animals (day 34 after SaOS-2 inoculation), all animals were euthanised under anaesthesia by cervical dislocation. The tumour-affected limbs were removed along with lungs, heart, intestines and skin. All specimens were fixed in 4% paraformaldehyde on harvesting. Tissues were embedded in paraffin prior to histological preparation and analysis. Four-*μ*m sections of lungs, heart, intestines, skin and primary tumours were cut by microtome. Both lungs and tumours were sectioned to achieve the greatest cross-sectional area for examination. The lungs, heart, intestine and skin sections were stained with haematoxylin and eosin. Apoptosis was assessed in sections of primary tumour using a terminal dUTP nick end-labelling (TUNEL) assay kit (Promega), according to the manufacturers’ instructions (Ta *et al*, 2009a). Blood sampling was performed immediately after cervical dislocation and dissection through the thoracic cage. Affected limbs were X-rayed at 35 kV for 30 s using a cabinet system (Faxitron Corp., Wheeling, IL, USA).

### Statistical and imaging software

GraphPad Prism 5 for Mac OS X (Version 5.0d) was used for all statistical tests. Student's *t*-test and ANOVA analysis with Bonferroni multiple comparisons test were used where appropriate. ImageJ (Version 1.45j, National Institutes of Health, USA) was used for all image analysis.

## Results

### PEDF induces apoptosis and inhibits cell cycling of osteosarcoma cells *in vitro*

*In vitro* studies were first performed in order to characterise the biological effects of PEDF on the SaOS-2 and SJSA-1 osteosarcoma cell lines. Cell viability was assessed by MTS proliferation assay, apoptosis by TUNEL assay and cell cycling by Ki-67 immunocytochemistry. The SaOS-2 cell line was used for the orthotopic murine model of osteosarcoma.

**Figure 1:**
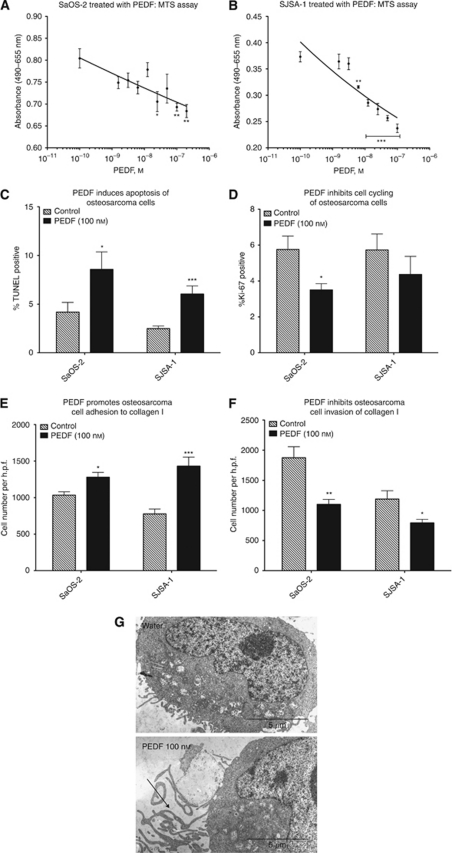
Biological effects of PEDF on SaOS-2 and SJSA-1 osteosarcoma cell lines *in vitro*. (**A** and **B**) MTS proliferation assay; (**C**) TUNEL assay; (**D**) Ki-67 immunocytochemistry; (**E**) collagen I adhesion assay; and (**F**) collagen I invasion assay. ^*^P<0.05, ^**^P<0.01 and ^***^P<0.001, two-tailed *t*-test (±s.e.m.). (**G**) Ultrastructural features of SaOS-2 osteosarcoma cells *in vitro*. Note the prolonged cell surface processes (arrow). h.p.f.= high power field.

Cell lines SaOS-2 and SJSA-1 were treated with PEDF at 1.56, 3.125, 6.25, 12.5, 25, 50 and 100 nM concentrations. The viability of SaOS-2 and SJSA-1 cells was reduced by 13.8% (*P*<0.01) and 36.4% (*P*<0.001, one-way ANOVA with Bonferroni multiple comparisons test, *n*=4, representative experiment), respectively, when treated with 100 nM PEDF (Figures 1A and B).

For the SaOS-2 cell line, 4.18% of control cells were identified as undergoing apoptosis by TUNEL staining. With PEDF treatment 8.57% were TUNEL positive, representing a two-fold increase in apoptotic cells (*P*<0.05, two-tailed *t*-test, *n*=4, four experiment repeats). Overall, 6.04% of SJSA-1 cells treated with PEDF were TUNEL positive, compared with 2.48% of cells that received the vehicle solution. This two-fold increase in apoptotic cells was also significant (*P*<0.001, two-tailed *t*-test, *n*=4, four experiment repeats; Figure 1C).

SaOS-2 osteosarcoma cells treated with PEDF exhibited reduced staining for Ki-67. Overall, 5.76% of control SaOS-2 cells stained positively for Ki-67. Following 48 h of PEDF treatment, 3.51% of SaOS-2 cells were Ki-67 positive. Treatment of SaOS-2 cells with PEDF resulted in a 60% reduction of cycling cells (*P*<0.05, two-tailed *t*-test, *n*=4, four experiment repeats). In total, 4.36% of SJSA-1 cells treated with PEDF stained positively for Ki-67, compared with 5.73% of the control cells (*P*=0.32, two-tailed *t*-test, *n*=4, four experiment repeats; Figure 1D).

Scanning electron microscopy demonstrated chromatin condensation within the nuclei of PEDF-treated SaOS-2 cells. Treated cells also showed deranged mitochondrial architecture and prominent cell surface processes (Figure 1G). Chromatin condensation and deranged mitochondria are consistent with osteosarcoma cells undergoing apoptosis. The significance of the cell surface processes remains unknown; however, one might speculate as to a possible role in cell–matrix or cell–cell adhesion.

### PEDF reduces the metastatic potential of osteosarcoma cells *in vitro*

The effect of PEDF on the metastatic potential of osteosarcoma cells was assessed *in vitro* by collagen I adhesion and invasion assays. Treatment with PEDF significantly promoted osteosarcoma cell adhesion to type I rat-tail collagen (Figure 1E). The result was most striking for the SJSA-1 cell line, which demonstrated an 83.9% enhancement in adhesion to the freshly set collagen (*P*<0.001, two-tailed *t*-test, *n*=2, three experiment repeats). For SaOS-2, treatment with PEDF improved adhesion by 23.9% (*P*<0.05, two-tailed *t*-test, *n*=2, three experiment repeats).

While enhanced adhesion to collagen I was striking for the SJSA-1 cell line with PEDF treatment, both cell lines in this experiment showed similar degrees of inhibition of invasion through collagen I (Figure 1F). There was a 41.3% reduction in the ability of SaOS-2 cells to migrate through the membrane (*P*<0.01, two-tailed *t*-test, *n*=2, two experiment repeats), and a 33.4% reduction for SJSA-1 cells (*P*<0.05, two-tailed *t*-test, *n*=2, two experiment repeats).

### Systemically administered PEDF inhibits growth of orthotopic osteosarcoma *in vivo*

Treatment with PEDF was delayed until day 20 after intra-tibial inoculation with the SaOS-2 human osteosarcoma cell line. Tumours were well established and macroscopically evident prior to initiating treatment protocols, thus replicating the human situation. The average tumour volume at this time was 21.1 mm^3^ (±2.357 s.e.m., *n*=12).

**Figure 2:**
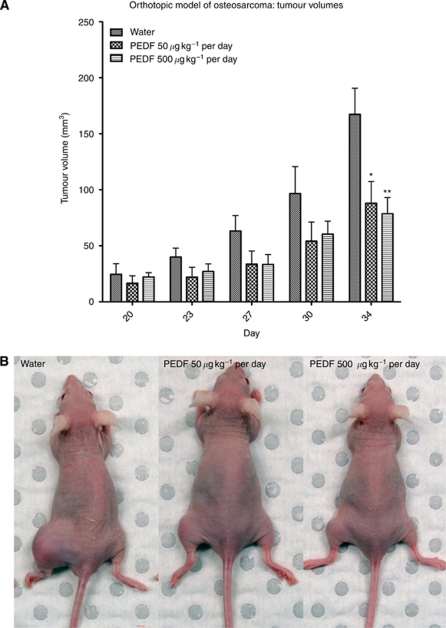
(**A**) Treatment with 50 and 500 *μ*g kg^−1^ per day PEDF doses caused 47.4% and 53% reductions in tumour volume at day 34, respectively. ^*^P<0.05, ^**^P<0.01, two-way ANOVA (±s.e.m.). (**B**) Photomicrographs of mice at day 34 showing orthotopic tumours involving left hindlimbs.

A surgically implanted intraperitoneal osmotic pump delivered PEDF. Sustained delivery of PEDF at both 50 and 500 *μ*g kg^-1^ per day doses caused a significant reduction in tumour volume by the study end point (Figures 2A and B). Animals treated with 50 *μ*g kg^−1^ per day PEDF dose exhibited a mean reduction in tumour volume of 47.4% at day 34 (*P*<0.05, two-way ANOVA with Bonferroni multiple comparisons test). The higher 500 *μ*g kg^−1^ per day PEDF dose caused a 53.0% reduction in tumour volume at day 34 (*P*<0.01, two-way ANOVA with Bonferroni multiple comparisons test). Day 34 was the humane end point of the study as tumours had grown to a disabling size. There was no statistical difference between groups receiving these two doses of PEDF.

**Figure 3:**
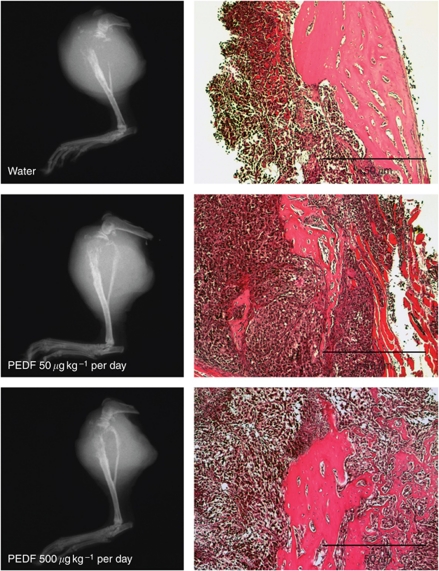
Plain radiographs of tumour-bearing limbs (left) and haematoxylin- and eosin-stained sections of primary tumour involving bone (right). Significant osteolysis and soft tissue invasion was seen for both control and treatment groups.

Orthotopic tumours were examined histologically for extent of invasion of surrounding structures, tumours necrosis and apoptosis. Treatment groups were unable to be differentiated based on these parameters. All animals showed extensive tumour invasion of soft tissue and bony structures. Specifically, tumour cells were seen within skeletal muscle, crossing the proximal physeal plate of the tibia and destroying normal bone architecture. In some cases tumours progressed to replace the distal femoral diaphysis. Plain radiographs of tumour-bearing limbs showed extensive soft tissue invasion and osteolysis for both treatment groups (Figure 3).

Orthotopic tumour tissue was sectioned to achieve a maximal *en face* surface for quantification of tumour necrosis and apoptosis. Haematoxylin- and eosin-stained sections were used to quantify tumour necrosis. Tumours treated with 50 *μ*g kg^−1^ per day PEDF dose showed 57.5% mean tumour necrosis, whereas those treated with 500 *μ*g kg^−1^ per day PEDF dose showed 31.5% mean tumour necrosis. Control tumours showed 28.2% tumour necrosis. There was no statistical significance between groups.

Adjacent sections of tumour were TUNEL stained and again there was no statistical significance between treatments based on % TUNEL-positive staining. Tumours from control, 50 and 500 *μ*g kg^−1^ per day PEDF dose groups demonstrated 20.2%, 45.9% and 21.2% TUNEL-positive tumour tissue, respectively.

### PEDF restricts progression of pulmonary metastatic disease

The burden of pulmonary metastatic disease at the study end point was assessed histologically on haematoxylin- and eosin-stained sections of lung tissue. Lungs were sectioned in order to achieve maximal cross-sectional area for study. At × 20 magnification, there was no significant difference in the mean number of pulmonary micrometastases observed between treatment groups. Control animals showed 7.5 micrometastases per lung section, as compared with 5.25 and 7.25 micrometastases for animals treated with 50 and 500 *μ*g kg^−1^ per day PEDF doses, respectively.

**Figure 4:**
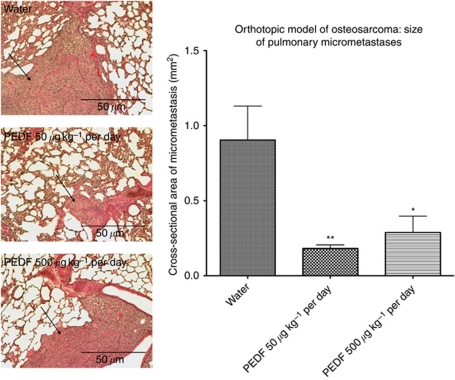
Treatment with 50 and 500 *μ*g kg^−1^ per day PEDF doses caused 79.8% and 68.1% reductions in mean cross-sectional area of pulmonary micrometastatic lesions. ^*^P<0.05, ^**^P<0.01, one-way ANOVA (±s.e.m.). Pulmonary metastases (arrows) were observed around larger airways and at the lung periphery (left).

The cross-sectional area of pulmonary micrometastases was measured. Ten micrometastatic lesions per treatment group were used in this analysis. Treatment with PEDF at 50 and 500 *μ*g kg^−1^ per day doses caused 79.8% (*P*<0.01) and 68.1% (*P*<0.05) reductions in mean cross-sectional area of micrometastatic lesions (one-way ANOVA analysis with Bonferroni comparison test). The mean area of pulmonary micrometastatic lesions was 0.90, 0.18 and 0.29 mm^2^ for control, 50 and 500 *μ*g kg^−1^ per day PEDF dose groups, respectively (Figure 4).

### Therapeutic safety of systemic PEDF

In addition to establishing the therapeutic efficacy of PEDF using the orthotopic model of osteosarcoma, we also sought to identify possible side effects associated with PEDF administration. No significant difference in animal weight was observed between PEDF-treated and control groups. Furthermore, the cachectic trend usually seen with this orthotopic model of disease was notably absent. At day 34, euthanasia was required, as tumours had grown to a disabling size in control animals.

Serum obtained at the study end point was analysed for renal and hepatic biochemical parameters. There was no significant difference between treatment groups, with serum creatinine, alkaline phosphatase and aspartate transaminase remaining within physiological limits.

Cardiac, small intestine and skin tissues were stained with haematoxylin and eosin and examined for signs of chemotherapy-associated toxicity. Treatment with conventional cytotoxic agents, such as doxorubicin, may be associated with the vacuolisation of myocardium and intestinal epithelium (Ta *et al*, 2009b). The lamina propria may become separated from the overlying intestinal and cutaneous epithelium, with loss of hair follicles (Tan *et al*, 2010). None of these changes were evident in either the PEDF-treated or control groups.

## Discussion

In this study we sought to evaluate the potential of PEDF as a sole treatment agent for advanced osteosarcoma. As an endogenous glycoprotein, PEDF is an attractive therapeutic agent in terms of potential chemoresistance and immunoreactivity. For the first time, we have successfully demonstrated a therapeutic effect for PEDF protein on both established primary osteosarcoma and pulmonary metastases. Additionally, we observed no adverse physiological effects associated with PEDF treatment.

Treatment with PEDF was delayed until orthotopic osteosarcoma was macroscopically evident, and despite this late stage of intervention, we observed 47.4% and 53.0% reductions in tumour volume by the study end point for 50 and 500 *μ*g kg^−1^ PEDF, respectively. Ek *et al* (2007a) showed an effect when 25 nM PEDF was co-administered at the time of orthotopic inoculation. Tumour volume and growth rates were reduced by 40%. We add clinical relevance to these findings by delaying treatment to better replicate the human presentation of disease. In another study, Ek *et al* (2007b) demonstrated a 51% reduction in tumour size when PEDF overexpressing SaOS-2 cells were used for intra-tibial injection. Ta *et al* (2009a) tested a chitosan hydrogel delivery system for PEDF plasmid. Treatment with Chi/DPO7-pPEDF resulted in a 37% reduction in tumour volume. Similarly, Dass *et al* (2006) used chitosan microparticles encapsulating PEDF plasmid for a therapeutic effect. All of these studies used the same SaOS-2 orthotopic model of osteosarcoma and are thus comparable. By using recombinant protein, rather than gene therapies that have yet to be successfully adopted for human disease, we have gone one step closer to mirroring the human condition.

The molecular mechanisms that PEDF uses to inhibit growth of osteosarcoma are yet to be fully elucidated and represent an important area for further research if we are to fully understand the therapeutic effects that the aforementioned studies have demonstrated. Pigment epithelium-derived factor has been shown to inhibit osteosarcoma growth both directly and indirectly. As we have shown here *in vitro*, direct inhibition occurs by both the induction of apoptosis and the inhibition of cell-cycle progression. Takenaka *et al* (2005) showed increased caspase-3/7 activity and decreased DNA synthesis by thymidine incorporation studies when MG63 osteosarcoma cells were treated with 100 nM PEDF. Ek *et al* (2007a) showed PEDF to induce apoptosis using UMR 106-01 and SaOS-2 cell lines by TUNEL assay.

Intriguingly, in our study we were unable to show a differential effect on either tumour necrosis or apoptosis *in vivo* with PEDF treatment. By allowing tumours to advance to palpable proportions prior to initiating treatment, we have potentially allowed them to outgrow their vasculature and so undergo spontaneous necrosis and apoptosis. Additional work is needed to clarify these processes *in vivo*; however, this should not detract from the finding that tumour volumes were reduced in PEDF-treated mice.

Animals that received PEDF treatment had a reduced burden of pulmonary metastatic disease at the study end point and this is in keeping with the findings of previous studies. We found the cross-sectional area of pulmonary metastases to be 79.8% and 68.1% smaller in animals receiving 50 and 500 *μ*g kg^−1^ PEDF doses, respectively. Ek *et al* (2007a) showed a 70% reduction in the mean number of macroscopic metastases when PEDF was co-administered at the time of orthotopic inoculation. When PEDF overexpressing SaOS-2 cells were used, no pulmonary metastases were observed (Ek *et al*, 2007b). Dass *et al* (2007) and Ta *et al* (2009a) demonstrated 2- and 8-fold reductions in the number of pulmonary metastases when applying chitosan microparticles and a hydrogel delivery system, respectively, to deliver PEDF plasmid.

Although the gross burden of pulmonary metastatic disease appeared to be reduced, we sought to further clarify the effect of PEDF on the metastatic process. Quan *et al* (2002) first showed that PEDF expression in the avascular zones of the growth plate was likely to inhibit invasion across epiphyseal cartilage. Ek *et al* (2007a) demonstrated dose-dependent reductions in adhesion and invasion of collagen I using the SaOS-2 cell line. We have replicated these findings and extended them to include the SJSA-1 cell line. Ek *et al* (2007a) also described a change in cellular morphology with PEDF treatment. With TEM we support these findings, as PEDF-treated SaOS-2 cells showed chromatin condensation, deranged mitochondrial architecture and prominent cell surface processes.

In our study, however, all tumours, irrespective of treatment, were found to be aggressively replacing local bony architecture and invading the surrounding soft tissues and musculature. When sections of lung tissue were examined under × 20 magnification, there was no difference between treatment groups in the number of observed micrometastases. When one considers the dramatic effect of PEDF on the size of pulmonary metastases, it is possible that systemic delivery of PEDF may not only inhibit the metastatic cascade, as demonstrated in previous studies, but may also have a direct effect on proliferation in pulmonary metastases. In order to further clarify the differential contributions of PEDFs anti-metastatic and anti-proliferative effects, an animal model that exploits real-time imaging of metastases would be of clear benefit.

Further work is needed to clarify the mechanisms of metastasis inhibition in osteosarcoma. Guan *et al* (2004) showed that PEDF-decreased invasion of malignant U251 glioma cells was related to downregulation of matrix-metalloproteinase-9 (MMP-9), an important enzyme for matrix degradation. Kozaki *et al* (1998) showed that PEDF secreted by colon cancer cells bound with high affinity to both collagen I and III and that expression of PEDF was inversely related to its metastatic capacity. The interactions between PEDF, MMPs and collagens, and their role in the metastasis of osteosarcoma have yet to be fully evaluated.

The use of an endogenous glycoprotein such as PEDF offers a number of advantages, such as reduced immunoreactivity and chemoresistance. Pigment epithelium-derived factor may be considered a targeted therapy for osteosarcoma, interacting specifically with the deregulated pathways of malignant cells, however as a physiological agent it is also critical for physiological process such as tissue healing and homeostasis. For the first time, our results not only confirm a therapeutic effect but also show PEDF to be a safe treatment. Serum and tissue analysis showed no evidence of toxicity, and animals remained well for the duration of the study.

In conclusion, this study provides evidence for PEDF as a therapeutic agent for osteosarcoma. Systemic PEDF restricts growth of both primary osteosarcoma and pulmonary metastases when treatment is delayed until after tumours become clinically palpable. We have optimised an established animal model and used recombinant PEDF to add clinical relevance to the findings. Our results also identify areas in need of further work. Studies are required to characterise the molecular mechanisms of PEDFs anti-osteosarcoma and anti-metastatic activity. Particularly, the use of real-time imaging would be beneficial in order to characterise the role of PEDF in the metastatic process.
